# Directing the migration of serum-free, *ex vivo*-expanded Vγ9Vδ2 T cells

**DOI:** 10.3389/fimmu.2024.1331322

**Published:** 2024-02-29

**Authors:** Kiran K. Parwani, Gianna M. Branella, Rebecca E. Burnham, Andre J. Burnham, Austre Y. Schiaffino Bustamante, Elisabetta Manuela Foppiani, Kristopher A. Knight, Brian G. Petrich, Edwin M. Horwitz, Christopher B. Doering, H. Trent Spencer

**Affiliations:** ^1^ Cancer Biology Program, Graduate Division of Biological and Biomedical Sciences, Emory University, Atlanta, GA, United States; ^2^ Aflac Cancer and Blood Disorders Center, Department of Pediatrics, Emory University School of Medicine and Children’s Healthcare of Atlanta, Atlanta, GA, United States; ^3^ Expression Therapeutics LLC, Tucker, GA, United States

**Keywords:** gamma delta T cells, gamma delta T cell migration, bone marrow, blood–bone marrow barrier, cancer immunotherapy

## Abstract

Vγ9Vδ2 T cells represent a promising cancer therapy platform because the implementation of allogenic, off-the-shelf product candidates is possible. However, intravenous administration of human Vγ9Vδ2 T cells manufactured under good manufacturing practice (GMP)-compliant, serum-free conditions are not tested easily in most mouse models, mainly because they lack the ability to migrate from the blood to tissues or tumors. We demonstrate that these T cells do not migrate from the circulation to the mouse bone marrow (BM), the site of many malignancies. Thus, there is a need to better characterize human γδ T-cell migration *in vivo* and develop strategies to direct these cells to *in vivo* sites of therapeutic interest. To better understand the migration of these cells and possibly influence their migration, NSG mice were conditioned with agents to clear BM cellular compartments, i.e., busulfan or total body irradiation (TBI), or promote T-cell migration to inflamed BM, i.e., incomplete Freund’s adjuvant (IFA), prior to administering γδ T cells. Conditioning with TBI, unlike busulfan or IFA, increases the percentage and number of γδ T cells accumulating in the mouse BM, and cells in the peripheral blood (PB) and BM display identical surface protein profiles. To better understand the mechanism by which cells migrate to the BM, mice were conditioned with TBI and administered γδ T cells or tracker-stained red blood cells. The mechanism by which γδ T cells enter the BM after radiation is passive migration from the circulation, not homing. We tested if these *ex vivo-*expanded cells can migrate based on chemokine expression patterns and showed that it is possible to initiate homing by utilizing highly expressed chemokine receptors on the expanded γδ T cells. γδ T cells highly express CCR2, which provides chemokine attraction to C-C motif chemokine ligand 2 (CCL2)-expressing cells. IFNγ-primed mesenchymal stromal cells (MSCs) (γMSCs) express CCL2, and we developed *in vitro* and *in vivo* models to test γδ T-cell homing to CCL2-expressing cells. Using an established neuroblastoma NSG mouse model, we show that intratumorally-injected γMSCs increase the homing of γδ T cells to this tumor. These studies provide insight into the migration of serum-free, *ex vivo-*expanded Vγ9Vδ2 T cells in NSG mice, which is critical to understanding the fundamental properties of these cells.

## 1 Introduction

Human γδ T cells represent 1%–5% of all T lymphocytes (1). γδ T cells, which diverge from their αβ T-cell counterpart that comprises 65%–70% of T cells, were discovered by their variant (V) γ chain of the T-cell receptor (TCR) (2). Variations of the γ and δ chains generate different subsets of γδ T cells (3, 4) with Vδ1, Vδ2, and Vδ3 as the main subsets of γδ T cells. Vδ1 and Vδ3 are abundant in the gut mucosa, whereas Vδ2 makes up the Vγ9Vδ2 subset and is the most prominent in circulation (5). Within the peripheral blood (PB), Vγ9Vδ2 T cells represent 60%–95% of the γδ T cells (6) and are considered part of both the innate and adaptive immune systems (7). They possess potent antitumor activity, which includes the inhibition of cancer cell proliferation and angiogenesis and the promotion of cancer cell apoptosis (8). An important feature of these cells is their TCR recognition of phospho-antigens (pAg) that accumulate in cancer cells through the dysregulation of the mevalonate pathway. Additionally, γδ T cells are not restricted to major histocompatibility complex (MHC)-mediated antigen presentation and thus do not require priming to recognize and kill targeted cells (9–11).

It has been demonstrated that γδ T cells can migrate *via* their chemoattractant properties (9, 12). Activated Vδ2^+^ T cells upregulate C-C chemokine receptors CCR1 and CCR2 and C-X-C chemokine receptor CXCR3, among others, and migrate to their respective, secreted chemokine ligands (13, 14). Many cells including epithelial cells (15), mesenchymal stromal cells (MSCs) (16), T cells (17), and tumor cells (18) have been shown to secrete the chemokine for CCR2, C-C motif chemokine ligand 2 (CCL2). Importantly, the CCR2/CCL2 axis has also been implicated in mobilizing cells to and from the bone marrow (BM) and to sites of inflammation (19). Chemokine analysis in melanoma samples prior to treatment identified elevated CCL2, which corresponded with increased T-cell migration and positive response to treatment (20). Another chemokine receptor, CXCR4, is highly expressed in CD4^+^ and CD8^+^ T cells (21), and CXCR4 mRNA is moderately expressed in γδ T cells (22). The ligand for CXCR4, CXC motif ligand 12 (CXCL12), is expressed by various types of stromal cells such as the skin, lymph nodes, and the BM (23). BM inflammation increases the secretion of CXCL12, which augments T-cell co-stimulation, proliferation, cytokine production, and migration (24). CD4^+^ and CD8^+^ T cells have been shown to home to inflamed BM based on the strength of the CXCR4/CXCL12 axis (21). However, the migration of γδ T cells is not as well understood.

*Ex vivo*-expanded γδ T cells are manufactured by stimulating peripheral blood mononuclear cells (PBMCs) with various agents including cytokines and aminobisphosphonates (ABPs) (25–27). γδ T cells require growth factors to expand, and the cytokines interleukin-2 (IL-2) and 15 (IL-15) are frequently added to culture conditions to promote γδ T-cell growth and enhance their antitumor properties (9, 28). Culturing with IL-15 significantly increases the expression of cytotoxic factors such as perforin and granzyme B, and IL-2 acts as a growth factor to increase γδ T-cell yield during expansion (29, 30). These cytokines are combined with ABP agents to further stimulate Vγ9Vδ2 T cells. ABP drugs inhibit the mevalonate pathway to produce the pAgs that activate butyrophilin in PBMCs, which stimulate the TCR of Vγ9Vδ2 T cells (31, 32). Zoledronate (zol) is a well-characterized ABP drug used alone or with IL-2 to activate Vγ9Vδ2 cells. Alternatively, synthetic ABPs, such as isopentenyl pyrophosphate (IPP) and IL-2, have been employed to expand Vγ9Vδ2 T cells (27, 33, 34). In addition, *ex vivo*-expanded Vγ9Vδ2 T cells can be engineered to express chimeric antigen receptors (CARs), which do not interfere with cellular innate killing or antigen-presenting capabilities (35), or bispecific T-cell engagers, for example, targeting CD19, a marker of B-cell malignancies, and have shown effective killing of CD19^+^ cell lines *in vitro* and *in vivo* (36, 37). In addition, non-signaling CARs were generated that activate alternate killing mechanisms of the engineered cells, such as through the receptor CD314 (NKG2D) (38).

We developed and optimized a good manufacturing practice (GMP)-compliant method of expanding and transducing or transfecting Vγ9Vδ2 T cells *ex vivo* (39, 40), which have been tested against glioblastoma, neuroblastoma, and T- and B-cell leukemias (22, 36, 41–43). The GMP-compliant expansion protocol is being tested in ongoing preclinical and clinical trials for several cancers. For example, a Phase I clinical trial is testing the combination of allogeneic Vγ9Vδ2 T cells with chemotherapy and the anti-GD2 antibody, dinutuximab, in relapsed or refractory neuroblastoma (NCT05400603). Although the preclinical data for these trials are extensive, they are confounded by the possible differences in partitioning of these cells within *in vivo* models, for example, mice, compared to the clinical setting. Several groups have shown that modified and non-modified cells are extremely effective *in vitro*; however, we routinely identify homing to the sites of malignancy as a limiting factor for *in vivo* therapeutic efficacy.

Despite their multi-faceted attributes, the Vγ9Vδ2 T-cell migratory phenotype *in vivo* has not been well defined, especially the migration pattern of serum-free expanded cells in NSG mice. Thus, the goal of this study was to better understand how *ex vivo*, serum-free-expanded Vγ9Vδ2 T cells function in NSG mouse models, particularly their migration and homing to the mouse BM, where systemically administered leukemias and lymphomas develop. Here, we employed various pharmacological agents, expansion protocols, and chemokine relationships to further elucidate the *in vivo* migration properties of Vγ9Vδ2 T cells and to induce cell migration to predetermined sites.

## 2 Materials and methods

### 2.1 Animal studies

All animal studies were conducted in accordance with the Emory University Institutional Animal Care and Use Committee (IACUC) regulations [protocol: PROTO201800202]. NOD.Cg-Prkdc^scid^IL2rg^tm1Wjl^/SzJ (NSG) mice (5–7 weeks old) were purchased from Jackson Laboratory (Bar Harbor, ME, USA) and housed in a pathogen-free facility. Where possible, equal numbers of male and female mice were used for all studies. γδ T cells were administered using retro-orbital injections, as this route has been well-characterized and shown to be as effective as tail vein injections (44, 45).

### 2.2 γδ T cells

γδ T-cell expansions were performed based on our previously published GMP-compliant protocol, which has been well-described and utilized in several publications from our group (22, 36, 39, 46). Whole blood was obtained from healthy donors through the Children’s Clinical Translational Discovery Core at Emory University, under approved Emory University Institutional Review Board (IRB) protocol, or from Expression Therapeutics LLC (Atlanta, GA, USA). PBMCs were isolated from fresh blood using Ficoll-Paque Plus (GE Healthcare Life Sciences, Milwaukee, WI, USA) density centrifugation. To preferentially select and expand γδ T cells, PBMCs were cultured in OpTmizer (Life Technologies, Carlsbad, CA, USA) containing OpTmizer supplement (Gibco, Grand Island, NY, USA), 1% penicillin/streptomycin (HyClone, Logan, UT, USA), and 2 mM l-glutamine (HyClone). Cells were then counted and resuspended at a concentration of 2e6 cells/mL in fresh culture media every 3 days. On days 0 and 3 of expansion, 5 µM zoledronate (Sigma-Aldrich, St. Louis, MO, USA) and 500 IU/mL IL-2 (PeproTech, Cranbury, NJ, USA) were added to the media. On day 6 of expansion, an αβ depletion was performed, described as previously published (47). Additionally, on days 6 and 9, 1,000 IU/mL IL-2 was added to the media. Expansion was ceased on day 12, and γδ T cells were used either fresh for experiments or frozen in PlasmaLyte A (Baxter International, Deerfield, IL, USA) containing 5% human serum albumin (Grifols, Barcelona, Spain) and 10% dimethyl sulfoxide (DMSO) (Sigma-Aldrich). Flow cytometry was performed on days 0, 6, and 12 to confirm successful expansion. Successful expansions resulted in cultures containing about 90% γδ T cells and less than 4% natural killer (NK) cells. The number of γδ T cells was determined by live cell counts multiplied by the percent of γδ T cells derived from flow cytometry (live CD3^+^ γδTCR^+^ cells). γδ T-cell fold expansion was determined by dividing the number of γδ T cells by the number on day 0 of expansion. Expansion results were analyzed on FlowJo software (v10).

### 2.3 Cell lines

Nalm6 and Nalm6-luciferase cells were a gift from the Porter Laboratory (Emory University). IMR5 cells were kindly provided by the Goldsmith Laboratory (Emory University). Nalm6 and IMR5 cells were cultured in RPMI 1640 (Corning, New York, NY, USA), 10% fetal bovine serum (FBS) (Bio-Techne, Minneapolis, MN, USA), and 1% penicillin/streptomycin (HyClone). CMK-luciferase cells were kindly provided by the Petrich Laboratory (Emory University) and were cultured with RPMI 1640, 20% FBS, and 1% penicillin/streptomycin.

### 2.4 Human MSCs

MSCs were isolated from BM in the residua (waste) of BM harvest collection bags obtained from healthy donors undergoing marrow harvest for clinical indications (Children’s Hospital Atlanta, Emory University). Where possible, equal numbers of male and female donors were used for each study. The protocol was classified as exempt from oversight by the Emory University IRB. MSCs were isolated by adherence to plastic cell culture plates (Corning), a method that has been well-documented and a standard for isolating MSCs (48). Cells were then expanded in culture with Dulbecco’s modified Eagle medium (DMEM) supplemented with 10% FBS, 1% penicillin/streptomycin, and 2 mM l-glutamine. In passage 2, our MSCs met the criteria proposed by the International Society for Cellular Therapy, and cells were maintained in culture at ∼60% confluence in media. Media were also supplemented with 1 ng/mL IFNγ (PeproTech) for 48 hours to create IFNγ-primed human MSCs (γMSCs). The resulting media were used for conditioned medium experiments.

### 2.5 Tissue collection and analysis

Mouse tissue collection was performed at the endpoint of each experiment. Mouse PB was collected using capillary tubes and deposited in tubes containing 10% ethylenediaminetetraacetic acid (EDTA) (Invitrogen, Carlsbad, CA, USA). Samples were centrifuged at 2,400 ×*g* for 15 minutes at 4°C. The plasma layer was discarded, and the remaining pellet was resuspended in 100 µL phosphate-buffered saline (PBS). Three red blood cell (RBC) lysis steps were performed by adding 3 mL RBC lysis buffer (Sigma), vortexing, and incubating at room temperature for 10 minutes. Samples were centrifuged at 300 ×*g* for 10 minutes, and the supernatant was discarded. After the last lysis, blood samples were resuspended in 100 µL PBS and stained for flow cytometry. Mouse BM was collected by harvesting femurs and tibias and cutting off the distal bone tips. A 23G needle (BD Horizon, Franklin Lakes, NJ, USA) was used to flush 1 mL PBS through the bone, and the marrow was collected. Samples were centrifuged at 300 ×*g* for 10 minutes. The supernatant was discarded, and the pellet was resuspended in 200 µL PBS. Samples were transferred to 0.35-µM cell strainers (Chemglass Life Sciences, Vineland, NJ, USA) on flow tubes. Tubes were centrifuged at 300 ×*g* for 10 minutes. The supernatant was discarded, and one RBC lysis was performed. After centrifugation at 300 ×*g* for 10 minutes, samples were stained for flow cytometry analysis.

### 2.6 Flow cytometry staining

When cells were ready for staining for flow cytometry, all samples were washed in flow tubes with 2 mL fluorescence-activated cell sorting (FACS) buffer. FACS buffer contains 2.5% fetal bovine serum (Bio-Techne) in PBS (Cytiva, Marlborough, MA, USA). Samples were centrifuged at 320 ×*g* for 3 minutes and decanted. Half the volume of “live/dead” control was removed and added to the “dead” tube, which was placed at 100°C for 2 minutes and then on ice for 2 minutes. Dead cells were added back to “live/dead” control. An antibody cocktail (all flow cytometry antibodies used in this study are listed in **Table 1**) was generated according to manufacturers’ dilution recommendations, along with BV buffer (BD Horizon), and 100 µL was added to each sample. One drop of UltraComp eBeads (Invitrogen), 1 µL antibody, and 100 µL FACS buffer were used as compensation controls. All tubes were covered and incubated for 20 minutes on ice and vortexed at 10 minutes. Then, 2 mL FACS buffer was added, and samples were centrifuged at 320 ×*g* for 3 minutes. The supernatant was decanted post-centrifugation, and flow cytometry was performed. Samples were run on the Cytek Aurora (Cytek Biosciences, Fremont, CA, USA) and analyzed using FlowJo. Mean fluorescence intensity (MFI) was calculated on FlowJo software (v10) and graphed on GraphPad Prism software (v10).

**Table 1:** All flow cytometry antibodies used in this study.

Marker (all human unless denoted otherwise)	Stain	Company	Catalog number	Clone/lot number
*CXCR4 #1	BV480	BD OptiBuild	746621	12G5/3009131
**CXCR4 #2**	APC	R&D Systems	FAB173A-025	44717
**CXCR4 #3**	AF 647	R&D Systems	FAB172R-100UG	44716
**CXCR4 #4**	APC-Vio 770	Miltenyi	130-116-667	REA649
**CCR2 #1**	BV711	BioLegend	357232	K036C2
**CCR2 #2**	BV786	BD OptiBuild	747855	LS132.1D9
*CD3	Spark Blue 550	BioLegend	344851	SK7/B311325
*CD45	BUV395	BD Horizon	563792	HI30/2017963
*mCD45	BV510	BioLegend	103138	30-F11/B360620
*γδTCR	PE	BD Biosciences	347907	11F2/2292377
*CD69	APC	BioLegend	310910	FN50/B268175
*CD335 (NKp46)	BV711	BD Horizon	563043	9E2/0321062
*TIGIT	APC-Fire750	BioLegend	372707	A15153G/B327004
*CD226 (DNAM-1)	BV605	BioLegend	338323	11A8/B310112
*CD56	APC-R700	BD Horizon	565139	NCAM16.2/1105545
*CD27	BV650	BD Horizon	564894	M-T271/9217315
*CD94	BV421	BD OptiBuild	743948	HP-3D9/1174031
*CD62L	PE-Cy7	BioLegend	304821	DREG-56/B288473
*CD314 (NKG2D)	PerCP-Cy5.5	BioLegend	320818	1D11/B308592

Antibodies denoted with an asterisk were used in the γδ T *in vivo* phenotype experiment.

### 2.7 *In vivo* conditioning experiment

NSG mice were conditioned with 25 mg/kg busulfan (Busulfex, DSM Pharmaceuticals, Durham, NC, USA) intraperitoneal injection, 300 µL incomplete Freund’s adjuvant (Millipore Sigma, Burlington, MA, USA) 1:1 with sterile PBS *via* intraperitoneal injection, or 1.5-Gy total body X-ray radiation (Rad Source RS 2000 Biological Research Irradiator). Twenty-four hours after conditioning, each mouse was retro-orbitally injected with 8e6 γδ T cells. PB and BM were collected after 24 hours and stained for flow cytometry with γδ TCR (BD Biosciences, San Jose, CA, USA), CD3 (BioLegend, San Diego, CA, USA), mCD45 (BioLegend), hCD45 (BD Horizon), and CXCR4 (BD OptiBuild). Results were analyzed on FlowJo software (v10).

### 2.8 *In vivo* radiation dosage experiment

NSG mice were conditioned with irradiation of 1.5 Gy or a split dose of 6 Gy (3 Gy at 4 hours apart). The protocol was then followed identically as the *in vivo* conditioning experiment described above.

### 2.9 *In vivo* cell tracking

BALB/cJ mice were bled, and blood was collected in tubes containing 10% EDTA. Cell Trace CFSE Cell Proliferation Kit (Thermo Fisher Scientific, Waltham, MA, USA) stock solution was prepared according to the manufacturer’s protocol by combining 18 µL DMSO to one vial of carboxyfluorescein succinimidyl ester (CFSE). This DMSO/CFSE solution was transferred to 20 mL of sterile PBS (CFSE/PBS solution). Blood samples were centrifuged at 250 ×*g* for 5 minutes, and the plasma layer was discarded. Cells were then resuspended in PBS, and 10e6 cells per mouse were counted. Cells were centrifuged at 250 ×*g* for 5 minutes. After centrifugation, cells were resuspended in the CFSE/PBS solution at a ratio of 10e6 cells to 5 mL CFSE/PBS. Then, cells and solution were incubated at 37°C shaking at 150 RPM for 20 minutes. Samples were then centrifuged at 250 ×*g* for 5 minutes. The supernatant was decanted, and cells were washed with 5 mL PBS and centrifuged again. After the last spin, the supernatant was decanted, and cells were resuspended at a concentration of 10e6 cells/100 µL PBS. CFSE-stained blood was loaded into insulin syringes and injected into NSG mice retro-orbitally 24 hours after mice were conditioned with a split dose of 6-Gy radiation, 3 Gy at 4 hours apart. Twenty-four hours after CFSE-blood transfusion, NSG mice were bled, and PB was collected into tubes containing 10% EDTA. Mice were euthanized, and femurs were collected; BM was harvested. PB samples and BM were prepared for flow cytometry according to the tissue collection protocol and stained with mCD45.1 (BD Biosciences), mCD45.2 (BioLegend), and TER119 (BioLegend). Flow cytometry was then performed, and results were analyzed on FlowJo software (v10).

### 2.10 γδ T-cell *in vivo* phenotype

NSG mice were conditioned with a split dose of 6-Gy radiation and observed for 48 hours. After conditioning for 24 hours, 8.2e6 γδ T cells were injected retro-orbitally. Twenty-four hours after T-cell injection, 100 µL PB and BM from both femurs and tibias were obtained from all mice. All cells from each condition were combined and stained for various T-cell markers for flow cytometry. Flow cytometry antibodies used in this experiment are denoted with an asterisk in **Table 1**. Flow cytometry was then performed, and results were analyzed on FlowJo software (v10).

### 2.11 Leukemia study

NSG mice were injected with 5e6 CMK-luciferase cells or 2e6 Nalm6-luciferase cells *via* the tail vein. Fifteen days post-inoculation, bioluminescent imaging was performed by injecting D-luciferin (PerkinElmer, Waltham, MA, USA) at a dose of 150 mg/kg *via* intraperitoneal injection 10 minutes prior to imaging. Images were then quantified using the IVIS Spectrum imaging system (PerkinElmer) for confirmation of cancer engraftment. On day 16, mice were then retro-orbitally injected with 1e6 γδ T cells. Twenty-four hours after the T-cell injection, the spleen and both femurs were harvested from mice. Cells were stained with markers for CMK, CD3^−^/CD33^+^ (BD Horizon), and Nalm6, CD19 (BD Horizon), as well as γδ T-cell markers. Flow cytometry was performed, and the results were analyzed on FlowJo software (v10).

### 2.12 Intraosseous MSC/γMSC study

MSCs were primed with 1 ng/mL IFNγ, and NSG mice were irradiated with 1.5-Gy total body irradiation (TBI) on the same day. Twenty-four hours later, 1.6e5 MSC or γMSCs were injected *via* intraosseous injection into the left femur of all mice. After 24 hours, 4e6 cells were injected retro-orbitally into all mice. Twenty-four hours post-γδ T-cell injection, femurs were harvested and stained for MSCs or γMSCs (CD90^+^/CD105^+^ both from BD Horizon) and γδ T cells. Flow cytometry was performed, and the results were analyzed on FlowJo software (v10).

### 2.13 Transwell migration

Polycarbonate 6.5-mm Transwell plates with a 3.0-µM pore (Corning Life Sciences) were used for transwell migration assays. In the lower chamber, 600 µL media and 500,000 γMSCs or non-primed MSCs were placed, and 500,000 γδ T cells were placed in the top chamber. Four hours after incubation, a Cellometer (Nexcelom, Lawrence, MA, USA) was used to count the number of γδ T cells that migrated to the bottom chamber. Then, the specific migration of cells to the bottom chamber was calculated using the equation below.


$$\begin{array}{l} \text{Specific migration}=\\ \frac{\;number\;of\;cells\;migrated\;to\;experimental\;media-number\;of\;migrated\;to\;control\;media}{total\;number\;cells\;added\;to\;top\;chamber}\\ . \end{array}$$

To confirm that CCL2 induced γδ T-cell migration to γMSCs, a transwell migration assay was performed using a CCL2 antibody (R&D Systems, Minneapolis, MN, USA; MAB679-500) blockade. In the bottom chamber, 600 µL media and 500,000 γMSCs were placed along with 5 µg or 10 µg CCL2 antibody. In the top chamber, 500,000 γδ T cells were placed. Four hours after incubation, the Cellometer was used to count the total number of γδ T cells that migrated to the bottom chamber.

### 2.14 CCL2 chemokine assay

Using the Proteome Profiler Human Chemokine Kit reagents (R&D Systems), IFNγ-primed and non-primed human MSC-conditioned media (24-hour incubation) were analyzed for the presence of secreted chemokines. To compare chemokine relative expression levels between activated and non-activated media, membrane blots were developed and imaged on Blue Devil X-ray autoradiography film (Genesee Scientific, El Cajon, CA, USA) on an X-ray film developer (MXR Imaging, San Diego CA, USA; SRX-101A) according to profiler kit manufacturer’s instructions.

### 2.15 γδ T-cell neuroblastoma model

This human neuroblastoma NSG mouse model has been previously published by our group as a bona fide model in which to study γδ T-cell characteristics in targeting cancer (41, 46). NSG mice were administered IMR5-luciferase cells subcutaneously. Tumors were measured using a caliper, and when they reached approximately 125 mm^3^ in volume, either 5e5 MSCs or γMSCs were injected intratumorally. Twelve hours after, γδ T cells labeled with 2.5e6 XenoLight DiR Fluorescent Dye (PerkinElmer) were infused *via* the tail vein, and migration throughout the animals was determined using the IVIS Spectrum imaging system (PerkinElmer). Isoflurane anesthesia (Piramal, Bethlehem, PA, USA) was used during imaging. Living Image software was used to acquire and analyze fluorescence and bioluminescence data and then to scale for analysis. Whole-body images were captured to determine the distribution of fluorescence throughout the body. To quantify the fluorescence at the site of the tumor, the lungs, head, and tail were physically covered to only capture images of the tumor, which were used to quantify tumor-specific fluorescence. Twenty-four hours post-γδ T-cell administration, tumors were harvested and stained for the presence of γδ T cells. Stained cells were processed *via* flow cytometry, and results were analyzed on FlowJo software (v10).

### 2.16 RNA sequencing

Two biological replicates of two different healthy donor PBMC samples were collected. γδ T cells were isolated from donor PBMC samples and expanded in serum free, *ex vivo* media. RNA was extracted from γδ T cells using the commercially available RNeasy Micro Kit (Qiagen, Valencia, CA, USA; 74004). Sequencing libraries were prepared using Illumina platforms. Samples were run on Illumina NovaSeq 6000 (instrument identifier number: HWI-ST1276) with a minimum of 20 million paired-end reads. Fastq files were mapped and aligned to GrCh38p13 and GenCode36 using Illumina Dragen v3.10.4a on Amazon Web Services. Quantification of aligned samples was achieved using salmon through Illumina Dragen v3.10.4a. Quantification files were imported into R using tximport, low counts were filtered out, and differential expression analysis was performed using DESeq2. Counts were averaged and normalized, and log2 of normalized counts was ascertained and plotted in a scatter plot using GraphPad Prism software (v10).

### 2.17 CCL2 ELISA

Using the Human MCP-1/CCL2 ELISA Kit (Millipore Sigma), IFNγ-primed and non-primed human MSCs were analyzed for the presence of secreted CCL2 24, 48, and 72 hours after priming. This assay was performed according to the manufacturer’s protocol. The quantitative γMSC CCL2 readout (pg/mL) was compared to the manufacturer’s standard curve and recorded at OD 450 nm using a spectrometer (Molecular Devices, San Jose, CA, USA; SpectraMax). CCL2 concentration was calculated and analyzed using GraphPad Prism.

### 2.18 Rigor of data/statistical analysis

All animal experiments were performed with a minimum of three biological replicates and in accordance with the Animal Use Alternatives (3Rs— reduction, refinement, and replacement). All *in vitro* studies were performed with a minimum of three biological replicates with the exception of the RNA-sequencing data and the chemokine blots of MSCs and γMSCs. However, these two pieces of data i) served as confirmation of previously published findings and ii) were supplemented by additional experiments, which are included in the manuscript. All statistical analyses were performed on GraphPad Prism, and each analysis method is provided in the figure legends. Results are presented as the mean ± standard deviation of the mean. Statistical significance is denoted by asterisks if p< 0.05.

## 3 Results

### 3.1 TBI enhances human γδ T-cell migration to murine bone marrow

**Figure 1:**
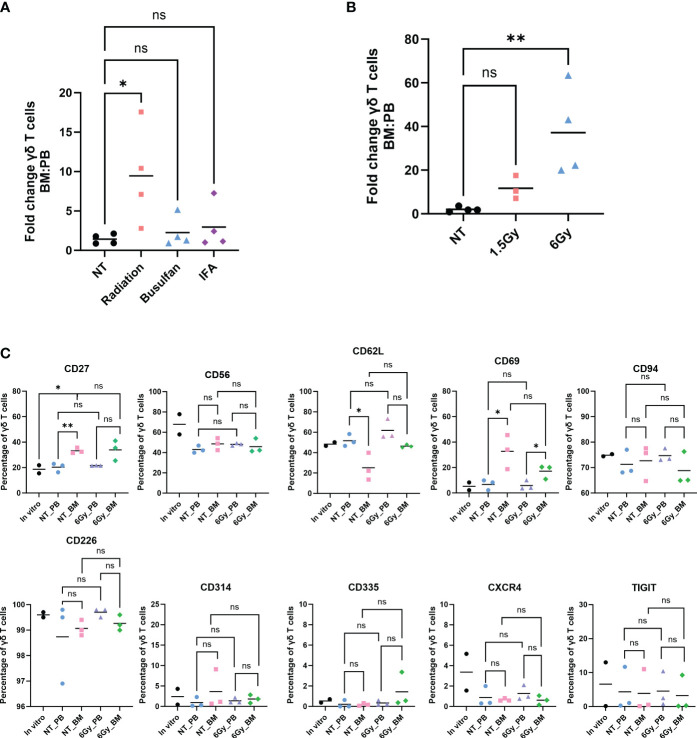
Human γδ T cells migrate to mouse bone marrow after radiation, and their phenotype is identical to that in the *in vitro-*expanded cells and circulation. Mice were conditioned with **(A)** 1.5-Gy radiation, 25 mg/kg busulfan, or 300 µL IFA (1:1 with PBS) or **(B)** 1.5 Gy or 6-Gy radiation; then, γδ T cells were administered, and 24 hours later, the percentage of γδ T cells was assessed by flow cytometry (gated on CD3^+^ γδTCR^+^ cells). **(A, B)** Statistics analyzed by non-parametric one-way ANOVA with *post hoc* (p< 0.05 = *); the sample mean is denoted with a black line; n = 3–4 mice per condition. **(C)** Mice were conditioned with 6-Gy radiation and 24 hours later injected with γδ T cells, and phenotype markers of live γδ T cells were assessed by flow cytometry. Each combination of samples was statistically analyzed by Student’s t-test (p> 0.05 = ns; p< 0.05 = *; p< 0.01 = **). ns, not significant. The *in vitro* combinations were all non-significant except for CD27. The sample mean is denoted with a black line. *In vitro* data represent two biological replicates; *in vivo* studies represent n = 3 mice for each condition. IFA, incomplete Freund’s adjuvant; PBS, phosphate-buffered saline.

We previously demonstrated that *ex vivo*, serum-free-expanded human Vγ9Vδ2 T cells, denoted herein as γδ T cells, do not persist in, or migrate to, murine BM (36). This is a concern because the NSG mouse is often used in preclinical testing of cell-based immunotherapies. Because γδ T cells migrate to sites of inflammation and tissue damage (49), in an attempt to enhance migration of human γδ T cells to murine BM, we conditioned NSG mice with low-dose TBI (1.5 Gy), incomplete Freund’s adjuvant (IFA), or 25 mg/kg busulfan, and we surveyed γδ T-cell percentages in the PB and BM. Radiation and busulfan are often used as conditioning agents to clear the BM compartment and initiate immune suppression in preparation for BM or mobilized hematopoietic stem cell transplants (50–52). IFA has been utilized in a prior study to boost the effectiveness of αβ T-cell migration to inflamed BM (21). Twenty-four hours after conditioning with these agents, we intravenously infused γδ T cells. Radiation significantly increased the relative percentage of human γδ T cells in the BM compared to the PB; IFA and busulfan did not show a significant difference in the relative percentages of γδ T cells in each compartment (**Figure 1A**). To further confirm that TBI increases the percentage of human γδ T cells in the BM, we conditioned mice with 1.5 Gy or 6 Gy and administered human γδ T cells. Conditioning mice with 1.5 Gy resulted in a minor increase in the percentage of γδ T cells, and 6 Gy significantly and dramatically increased this percentage (**Figure 1B**). Based on these findings, we used 6-Gy radiation for subsequent experiments.

We then evaluated if the γδ T cells entering the BM were phenotypically different than those remaining in circulation. We surveyed cell surface proteins using markers of γδ T-cell activation, inhibition, or enhancement of other specific properties: CD27, CD56, CD62L, CD69, CD94, CD226, CD314, CD335, CXCR4, and TIGIT. The expression of these markers was similar whether the cells were in circulation or the BM, with the exception of CD69, an activation marker of γδ T cells, which was elevated in cells in the BM (53) (**Figure 1C**). Importantly, the trend remained the same between the PB and BM of non-treated mice and irradiated mice. Furthermore, the γδ T-cell phenotype remained unchanged when comparing *in vitro* cultured cells to *in vivo* circulating cells obtained from the PB or BM, with the one exception of CD27. In addition, the distribution of cells with regard to each marker in the γδ T-cell population was similar when comparing cells in the PB to cells with those in the BM (**Supplementary Figure 1**). Therefore, of the conditions tested, TBI, busulfan, or IFA, TBI is an effective conditioning treatment to enhance migration of human γδ T cells into mouse BM, and the γδ T cells entering the BM are phenotypically unchanged from those cultured *in vitro*, in the PB, or in non-treated mice.

We next determined if human leukemia cells could provide a driving force to induce γδ T-cell migration into the BM. Prior studies from our lab using leukemia models demonstrated that γδ T cells, administered shortly after cancer inoculation in a mouse, do not traffic to the leukemic BM (36, 54). Therefore, the administered T cells do not target the BM-residing cancer, even if the T cells are engineered to express CARs against leukemia antigens. We employed a modified experimental design wherein we allowed time for two different luciferase-tagged, human leukemia cell lines (CMK, acute megakaryoblastic leukemia, and Nalm6, B-cell precursor leukemia) to completely engraft in the BM (**Supplementary Figure 2A**) before systemically infusing γδ T cells. Even under high leukemic stress, γδ T cells do not enter the BM despite the presence of human hematopoietic leukemia cells (**Supplementary Figure 2B**). Furthermore, we also did not observe an overall greater percentage of γδ T cells in the spleen (**Supplementary Figure 2C**). Therefore, *ex vivo*, serum-free-expanded γδ T cells do not traffic to the BM of mice even under a high leukemic burden.

### 3.2 Homing is not the mechanism of radiation-induced migration of γδ T cells into the bone marrow

**Figure 2:**
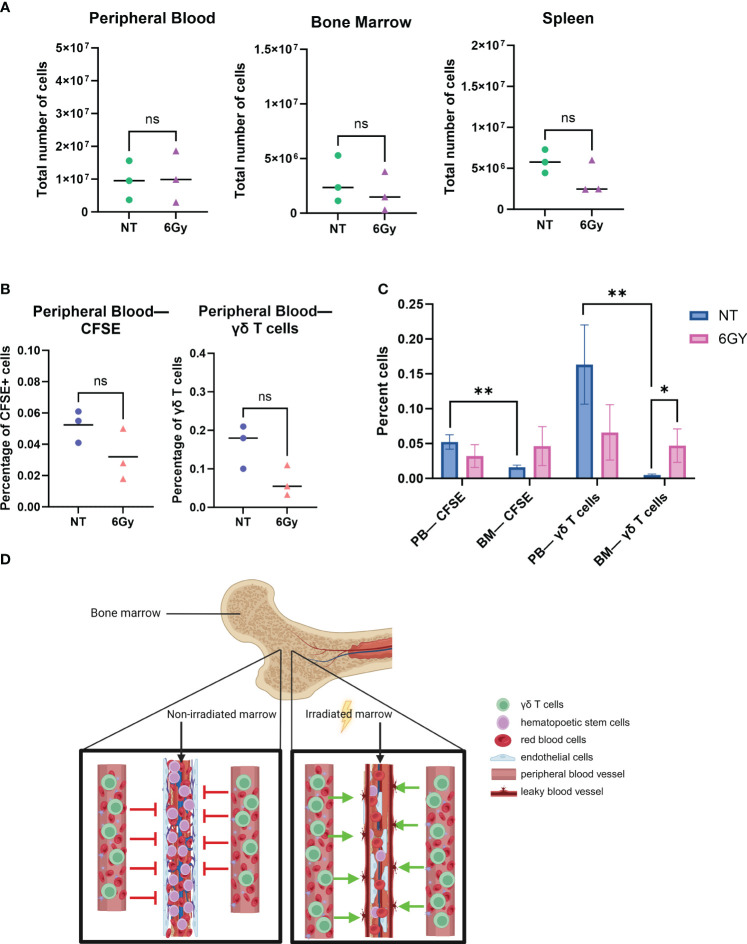
Radiation breaks down the blood–bone marrow barrier, allowing circulating γδ T cells to filter into and through the bone marrow space. **(A)** Mice were conditioned with 6-Gy radiation 24 hours prior to the injection of γδ T cells; 24 hours after administration of γδ T cells, blood, marrow, and spleen were harvested, and cells were counted with trypan blue. **(B)** Mice were conditioned with 6-Gy radiation, and then 24 hours later, they were injected with 10e6 γδ T cells or CellTrace CFSE-stained red blood cells from BALB/cJ mice; blood was collected and assessed for percentage of live CFSE-tagged cells (gated on CFSE^+^ TER119^+^ cells) or γδ T cells *via* flow cytometry. **(C)** Comparison of percentage CFSE+ cells and γδ T cells in non-treated or irradiated mouse blood and marrow. All experiments in this figure were performed with n = 3 mice per condition. All statistics analyzed by Student’s t-test (p< 0.05 = *; p< 0.01 = **), and the sample mean is denoted with a black line. **(D)** Graphical depiction of passive migration of circulating cells entering the bone marrow due to radiation-induced mechanical breakdown of blood–bone marrow barrier. CFSE, carboxyfluorescein succinimidyl ester. ns, not significant.

Although increased percentages of γδ T cells were observed in the BM of TBI-treated mice, we next determined if the γδ T cells were entering the BM due to a homing/trafficking axis or passively through the circulation. As shown in **Figure 1**, the phenotype of γδ T cells was similar in the BM and PB, which led us to hypothesize that these T cells do not home to the BM but instead passively flow into the BM from the circulation. To test this, we stained RBCs from BALB/cJ mice with CellTrace CFSE proliferation dye and systemically injected them into irradiated or non-irradiated NSG mice. We found that 6-Gy radiation did not significantly affect the total number of cells in the PB, BM, or spleen (**Figure 2A**), and radiation did not alter the percentage of CFSE^+^ RBCs or γδ T cells in the circulation (**Figure 2B**). Consistent with our previous results, we observed radiation significantly increased the percentage of γδ T cells in the BM (**Figure 2C**). In addition, when comparing CFSE^+^ RBCs or γδ T cells in non-treated mouse PB versus BM, we again observed a lower percentage of γδ T cells in the BM compared to PB. However, after radiation, the percentage of CFSE-marked RBCs was higher compared to that in non-treated mice and was similar to the percentage of marked cells in PB. Therefore, radiation allows CFSE^+^ RBCs to migrate freely through the BM, as the barrier that limits movement into the BM appears to be eliminated.

To demonstrate that TBI effects on the BM are quantitatively different than the effects of other agents, mice were conditioned with 25 mg/kg busulfan and then systematically injected with CFSE^+^ RBCs 24 hours later, and CFSE^+^ RBCs were surveyed in the PB and BM. The difference in the percentage of CFSE^+^ RBCs in the PB of non-treated mice compared to busulfan-treated mice was insignificant (**Supplementary Figure 3A**), and there was no significant difference in the percentage of CFSE^+^ RBCs in the BM of non-treated or busulfan-treated mice (**Supplementary Figure 3B**), a result different from that observed with radiation, as TBI significantly increased the percentage of γδ T cells in the BM (**Figure 2C**).

These data show that i) the γδ T-cell phenotype is the same when comparing cells harvested from PB or BM after *ex vivo-*expanded cells are administered to NSG mice; ii) the phenotype of the cells in circulation and in the BM is similar to that of cultured γδ T cells; iii) TBI, but not busulfan, resulted in an increase in the absolute number and percentage of γδ T cells in the BM; iv) similar to γδ T cells, there are fewer CFSE^+^ RBCs in BM compared to the PB, unless the mice are irradiated; and v) there was no difference in the percentage of CFSE^+^ RBCs or γδ T cells in the PB or BM when mice are conditioned with TBI. Therefore, because it is known that radiation induces the breakdown of the blood–BM barrier (55, 56), it is reasonable to conclude that TBI allows γδ T cells to passively flow through the marrow niche, as depicted in **Figure 2D**.

### 3.3 The lack of γδ T-cell homing to the BM is, in part, due to the absence of CXCR4 expression

**Figure 3:**
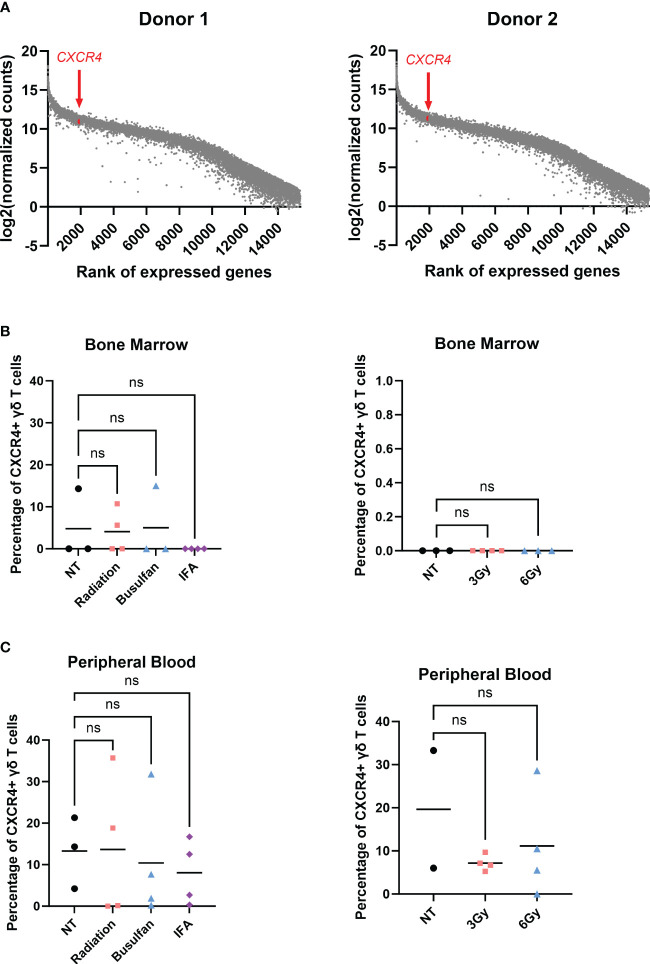
γδ T cells do not migrate to the bone marrow *via* the CXCR4/CXCL12 axis. **(A)** RNA-seq performed on two individual biological replicates of γδ T cells isolated from two different PBMC donors; genes were ranked, and log2(normalized counts) was calculated; *CXCR4* highlighted in red. **(B)** Mice were conditioned with 1.5-Gy radiation, 25 mg/kg busulfan, or 300 µL IFA (1:1 with PBS) or 3-Gy or 6-Gy radiation and then systemically infused with γδ T cells; 24 hours later, bone marrow and **(C)** blood were harvested and assessed for live CXCR4^+^ γδ T cells *via* flow cytometry using the CXCR4 BV480 antibody. For NT, n = 2–3 mice, and n = 4 for conditioned mice **(B, C)**. **(B, C)** Statistics analyzed by non-parametric one-way ANOVA with *post hoc* (p> 0.05 = ns), ns, not significant, and the sample mean is denoted with a black line. RNA-seq, RNA sequencing; PBMC, peripheral blood mononuclear cell; IFA, incomplete Freund’s adjuvant; PBS, phosphate-buffered saline.

**Figure 4:**
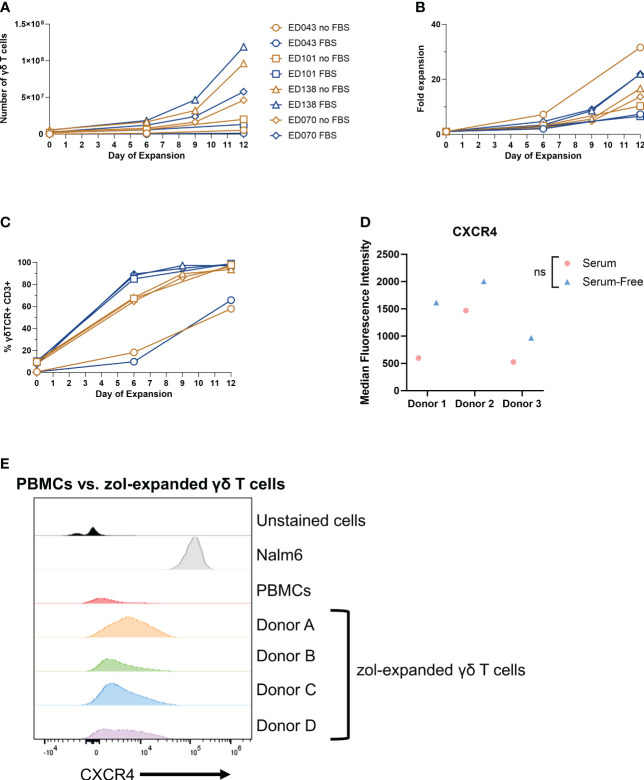
Low γδ T-cell CXCR4 expression is not due to serum-free expansion. Peripheral blood mononuclear cell samples from four individual healthy donors (n = 4 biological replicates) were selected for γδ T cells expanded in serum- or serum-free media. The following parameters were determined: **(A)** number of γδ T cells (live cell counts multiplied by percent γδ T cells derived from flow, CD3^+^ γδTCR^+^), **(B)** fold expansion (number γδ T cells divided by number on day 0), and **(C)** the percentage of γδ T cells by flow cytometry gated on γδTCR^+^ CD3^+^ cells. **(D)** CXCR4 mean fluorescence intensity (MFI) of γδ T cells expanded in serum- or serum-free media from three healthy peripheral blood mononuclear cells (n = 3 biological replicates) calculated in FlowJo software. Statistics analyzed by paired t-test (p > 0.05 = ns). ns, not significant. **(E)** Histogram of CXCR4^+^ γδ T cells in unstained γδ T cells, Nalm6 cells as a positive control, peripheral blood mononuclear cells, and four different zoledronate-expanded γδ T cells (n = 4 biological replicates).

When inflammation is induced in the BM, BM stromal cells release the chemokine CXCL12, or stromal-cell derived factor-1α (SDF-1α) (57, 58). BM-infiltrating αβ T cells highly express CXCR4, the G-protein-coupled receptor (GPCR) for CXCL12, and migrate to the BM based on CXCR4-CXCL12 chemoattraction (21). We show by RNA sequencing (RNA-seq) analysis that *CXCR4* mRNA was highly expressed in γδ T cells (top 6% of all RNA sequenced, **Figure 3A**), indicating that these cells should also migrate *via* the CXCR4/CXCL12 axis. Surprisingly, the percentage of CXCR4^+^ γδ T cells in the BM was very low regardless of conditioning regimen (**Figure 3B**), and there was a slightly higher percentage of circulating CXCR4^+^ γδ T cells (**Figure 3C**). Therefore, although the *CXCR4* mRNA was high in these γδ T cells, CXCR4 protein expression was low, which is consistent with previous studies (59, 60). To further characterize CXCR4 expression and determine if the lack of expression can be a consequence of serum-free expansion, γδ T cells were expanded from four donor PBMCs in either FBS media (SM) or serum-free media (SFM). The difference in the number of γδ T cells in each donor was non-significant in expanding in SM versus SFM, although there was the expected donor variability where some donors expanded better in one medium compared to the other (**Figure 4A**). Additionally, there was no change in the overall fold expansion (**Figure 4B**). The percentage of γδ T cells was higher in three out of the four donors expanded in SM on day 6 of expansion, but there was no significant difference in this percentage by the end of the expansion on day 12 (**Figure 4C**). Overall, we did not observe significant changes in major cell characteristics within the cellular product with the addition of serum to our expansion protocol.

To determine whether CXCR4 expression changes in γδ T cells expanded in SM or SFM, we evaluated the CXCR4 expression in γδ T cells expanded in zol from PBMCs from three different donors and cultured in either SM or SFM. All samples had low CXCR4 expression regardless of culturing media, but we noted a slight, non-significant increase in CXCR4^+^ γδ T cells from PBMCs expanded in SFM (**Figure 4D**). We confirmed these data using four different CXCR4 flow cytometry-confirmed antibody clones, with Nalm6 cells as a positive control (**Supplementary Figure 4A**). Furthermore, to determine if zol impacts CXCR4 expression, we measured CXCR4 by flow cytometry on γδ T cells from PBMCs and compared it to zol-expanded γδ T cells from four different donors. CXCR4 expression was similar in all samples, indicating that zol did not alter CXCR4 expression (**Figure 4E**).

Within these SF- or SFM-expanded cells, we also determined the percentage of NK cells, CD56^+^ γδ T cells, and CD16^+^ cells, which can be markers for enhanced cytotoxicity and improved antibody-dependent cellular cytotoxicity (61–63). The percentage of NK cells decreased in three of the four donor samples during expansion regardless of SM or SFM (**Supplementary Figure 4B**), and CD16 showed a minor increase at the end of expansion (**Supplementary Figure 4C**). Lastly, the percentage of CD56^+^ γδ T cells increased slightly or remained the same over the course of expansion, similar in SF or SFM (**Supplementary Figure 4D**). Therefore, SM does not appear to dramatically alter the phenotype of *ex vivo*-expanded γδ T cells.

Taken together, these data show that despite varying culturing and manufacturing conditions, γδ T cells express low levels of surface CXCR4, the level of which is likely insufficient to induce trafficking to the BM *via* the CXCR4/CXCL12 axis.

### 3.4 γδ T-cell homing can be directed by controlling chemokine expression

**Figure 5:**
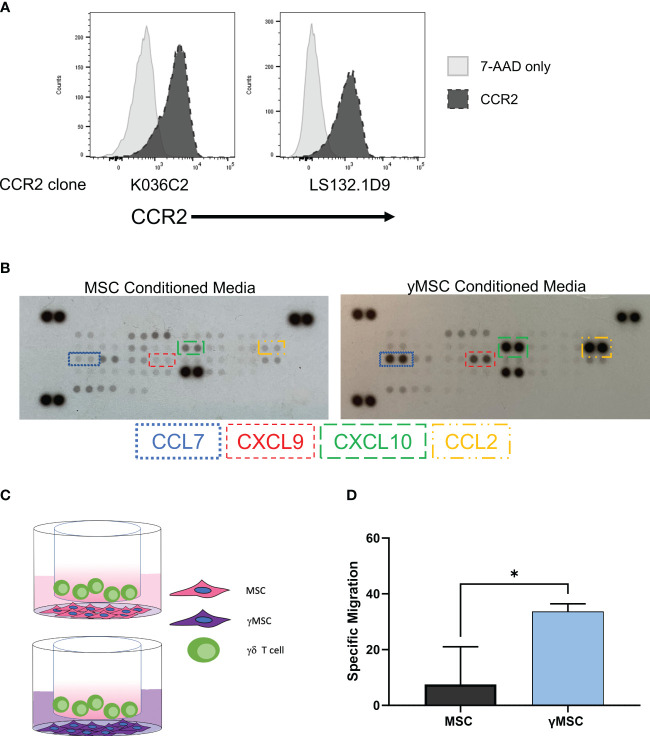
γδ T-cell homing can be influenced by leveraging the CCR2/CCL2 axis. **(A)** Flow cytometry histograms depicting CCR2 expression on γδ T cells with two different CCR2 antibody clones; 7-AAD was used as a live/dead control. **(B)** Representative human chemokine array membrane blot surveying secretion by MSCs from MSC- or γMSC-conditioned media. **(C)** Graphical depiction of the transwell migration assay with γδ T cells migrating to either MSCs or γMSCs with the respective conditioned media. **(D)** Specific migration of γδ T cells to MSCs or γMSCs in the transwell assay; n = 3 biological replicates, statistics analyzed by Student’s t-test (p< 0.05 = *), and error bars represent standard deviation. MSCs, mesenchymal stromal cells.

We showed that the chemokine receptor CXCR4 is not highly expressed on the surface of Vγ9Vδ2 cells, which we infer may be a mechanism for their poor migration to murine BM. This raised the concern as to whether or not these cells can migrate *via* other chemokine/chemokine receptor relationships. To determine if these *ex vivo*-expanded cells can migrate *via* receptor/ligand interactions and if these receptors can be leveraged to direct migration of γδ T cells to predetermined sites, we developed a model that conditionally expresses CCL2. We previously demonstrated that of the chemokine receptors expressed on the surface of serum-free-expanded γδ T cells, CCR2 showed the greatest expression (22). To confirm these results, we measured CCR2 expression with two different flow cytometry antibody clones (**Figure 5A**). Additionally, RNA-seq results using RNA isolated from γδ T cells from two different PBMC donors showed high *CCR2* expression, which was in the top 17% of RNA sequenced (**Supplementary Figure 5A**). Therefore, CCR2 had high protein expression correlating with high mRNA expression, unlike CXCR4.

It is well-established that CCR2 ligands include CCL2, CCL7, CCL8, and CCL13 with CCL2 considered to be the major ligand (64, 65). We developed a model to conditionally express CCL2, which takes advantage of chemokine expression differences when *ex vivo-*expanded MSCs are treated with interferon γ (IFNγ) to produce γMSCs. *Ex vivo-*expanded MSCs typically do not robustly express chemokines, but a human chemokine array demonstrated that γMSCs highly secrete CXCL9, CXCL10, CCL7, and CCL2 compared to non-primed MSCs (**Figure 5B**). CCL2 secretion was specifically monitored by enzyme-linked immunosorbent assay (ELISA), which showed that CCL2 significantly increased as early as 24 hours after priming and continued to increase for at least 72 hours (**Supplementary Figure 5B**). Therefore, γMSCs can be used to test the chemokine-induced migration ability of *ex vivo*, serum-free-expanded γδ T cells by capitalizing on the high CCL2 secretion after MSC priming.

**Figure 6:**
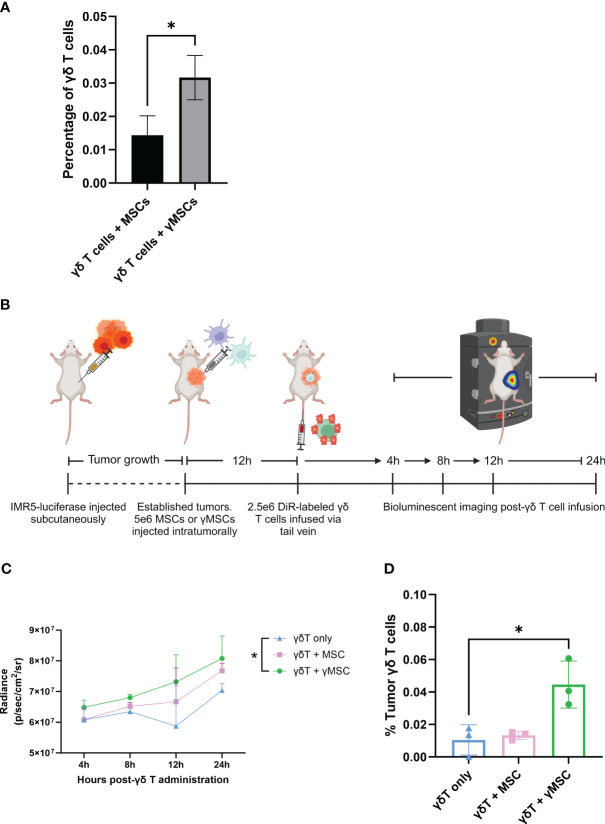
γδ T cells can be recruited to bone marrow and tumors through chemoattractant relationships. **(A)** NSG mice (n = 3 per condition) were conditioned with 1.5-Gy TBI 24 hours prior to intraosseous injection of 1.6e5 MSCs or γMSCs. After 24 hours, γδ T cells were injected retro-orbitally. Twenty-four hours later, femurs were harvested and stained for flow cytometry. The percentage of γδ T cells was then calculated. Error bars represent standard deviation. Statistical significance was analyzed by Student’s t-test (p< 0.05 = *). **(B)** Schematic of experiment. Mice were injected with IMR5-luciferase cells; tumors were established, and MSCs or γMSCs were injected intratumorally. n = 3 mice per condition. Twelve hours later, DiR-labeled γδ T cells were injected *via* the tail vein, and migration *in vivo* was monitored over 24 hours through relative bioluminescence. **(C)** Bioluminescent quantitative analysis of γδ T-cell trafficking to tumor over 24 hours. Statistics analyzed by paired t-test with Bonferroni correction (p< 0.05 = *). **(D)** Flow cytometry analysis of the percentage of γδ T cells per tumor 24 hours post-infusion. Statistics analyzed by paired t-test with Bonferroni correction (p< 0.05 = *), and error bars represent standard deviation. TBI, total body irradiation; MSCs, mesenchymal stromal cells.

To evaluate the migration of γδ T cells to γMSCs, a transwell migration assay was used with γδ T cells in the upper chamber and MSCs or γMSCs below (**Figure 5C**). Analysis of this assay showed significantly greater migration of the γδ T cells to the γMSCs than to the un-primed MSCs (**Figure 5D**). To further confirm that the trafficking is due to the CCR2/CCL2 axis, a transwell migration assay was performed, but a CCL2 blocking antibody was included to suppress CCL2 interaction with CCR2. Blocking CCL2 secretion significantly decreased γδ T-cell migration to γMSCs (**Supplementary Figure 5C**), indicating that these cells, indeed, show specific migration along this chemokine/receptor axis. To determine whether γδ T cells can significantly migrate to γMSCs *in vivo* as they do *in vitro*, MSCs or γMSCs were injected intrafemorally, and γδ T cells were administered systemically *via* retro-orbital infusion 24 hours later. γδ T cells were found to indeed migrate to γMSC BM significantly more than to MSC BM (**Figure 6A**).

To further investigate the chemoattraction properties of γδ T-cell migration, a neuroblastoma NSG mouse model was developed using IMR5-luciferase cells (**Figure 6B**), as we previously published (41, 46). IMR5 tumors were established in NSG mice, and MSCs or γMSCs were administered intratumorally. Then, DiR-labeled γδ T cells were administered intravenously 12 hours later. Tumor infiltration of γδ T cells was measured 24 hours later. Increased presence of γδ T cells was consistently observed in the tumors that harbor γMSCs (**Figure 6C**). In addition, there was no difference between the percentage of γδ T cells that infiltrated the tumor when no MSCs or non-primed MSCs were administered; however, there was a significant increase in the percentage of γδ T cells in the tumor when the tumors harbored γMSCs (**Figure 6D**). Taken together, these data demonstrate that *ex vivo*, serum-free-expanded γδ T cells can migrate along chemoattractant pathways, using chemokine receptors that, *a priori*, have been shown to be upregulated at the mRNA and protein levels.

## 4 Discussion

There is an unmet need in the field of γδ T-cell therapeutic development to better understand the fundamental properties of these cells, such as migration and trafficking, especially in diverse models of cancer. In this study, we characterized various aspects of serum-free, *ex vivo*-expanded human Vγ9Vδ2 T-cell migration in mice and explored the use of varying methods of directing the migration of these cells. Overall, we demonstrated that γδ T-cell migration can be influenced either through physical means, such as the use of TBI, or by utilizing chemokine expression profiling.

γδ T cells share many effector characteristics with αβ T cells such as cytokine production, cytotoxicity, and antigen presentation (49). Unlike αβ T cells, γδ T cells are not dependent on peptide/class II presentation and therefore can be used as an allogeneic treatment without causing graft-versus-host disease (GvHD) (66, 67). γδ T cells can also be significantly expanded and stimulated by different methods without compromising their antitumor properties (68). Indeed, we and others have documented the therapeutic potential of γδ T cells, and there are now several methods for expanding these cells under GMP conditions (22, 36, 38–43, 46). The foundation on which these cells can be employed as anti-cancer therapeutics has been well-documented. For example, a study surveying 18,000 tumors across 39 malignancies reported γδ T cells as the most prognostically favorable subset of tumor-infiltrating lymphocytes (TILs) (69). Additionally, a correlation was uncovered between the relative abundance of γδ TILs and favorable response to immune-checkpoint therapy in many cancers, highlighting an important combination therapeutic approach (6).

Although we and others demonstrated the cytotoxic potential of γδ T cells and an enhancement of target cell killing when engineered to express CARs targeting human cancers (36, 70, 71), a limiting aspect of using these cells is the lack of migration to the sites of tumors, such as the BM (36, 59). We surveyed varying agents that are known to affect the BM compartment. TBI and busulfan are experimentally and clinically used to clear the BM in preparation for BM transplants (51), and IFA is an adjuvant described to promote immune stimulation of αβ T cells (21). We demonstrated a significant increase in the percentage of γδ T cells entering the BM when mice are treated with TBI compared to other conditioning agents. Furthermore, our findings indicate that the increased migration of γδ T cells to the BM is the result of increased blood flow through the BM, which we postulate is caused by a TBI-induced breakdown of the blood–BM barrier. This phenomenon is important, as it has been shown that BM inflammation and breakdown enhance T-cell trafficking to the BM (72, 73); nevertheless, we show that mechanical breakdown of the blood–BM barrier causes passive migration from circulation and is the likely mechanism for the increased cellular accumulation.

Kuksin et al. found human CXCR4^+^ αβ T cells home to mouse BM *via* the chemoattractant relationship between CXCR4 and the highly secreted CXCL12 by the BM stromal cells caused by increased BM inflammation (21). We hypothesized that this relationship should apply to γδ T cells as well. However, despite RNA-seq showing high *CXCR4* mRNA expression, we found our *ex vivo*, SFM*-*expanded γδ T cells do not highly express CXCR4 protein on the cell surface. Our data are consistent with previous findings (59), and we can conclude that the lack of CXCR4 is at least one reason that these *ex vivo*, SFM-expanded γδ T cells do not traffic to the BM *via* the CXCR4/CXCL12 axis.

We also determined the low CXCR4 surface expression contrasted with its high mRNA expression. CXCR4 has been described in non-γδ T cells to undergo ligand-induced endocytosis, like other GPCRs, upon CXCL12 binding (74, 75). CXCR4 endocytosis is then followed by ubiquitination and degradation of the protein (76). Although this event has not been studied in γδ T cells, we initially thought that degradation of CXCR4 also occurs in γδ T cells after binding to CXCL12, which would explain why mRNA is high and surface protein is low. However, since these cells do not traffic to the BM and interact with the CXCL12 axis, this event cannot account for low CXCR4 levels in our γδ T cells. Nevertheless, this phenomenon is important for future studies to i) elucidate the mRNA–protein discrepancy and ii) engineer methods by which to increase extracellular CXCR4 and cause γδ T-cell trafficking to CXCL12. For example, recent studies have utilized γδ T-cell SFM containing the regulatory cytokine transforming growth factor (TGF) β to stimulate T cells and increase cytotoxicity and upregulation of chemokines (59, 77). TGF-β was shown to increase CXCR4 on some γδ T-cell expansions and promote migration to transformed cells. Beatson et al. demonstrated an increase in TGF-β-exposed γδ T-cell cytotoxicity of cell lines and significant clearing of cancer *in vivo* compared to cells expanded in ABP and IL-2 alone (59). Additionally, culturing γδ T cells in TGF-β instead of with IL-2 alone significantly increases the γδ T-cell migration to the BM (59).

Although the use of TGF-β has been documented for enhancing the cytotoxic activity and chemokine receptor expression of γδ T cells, it has also been well-characterized as a promoter of cancer cell epithelial-to-mesenchymal transition (EMT), cell proliferation, and evasion of immune surveillance and is not uniformly effective in γδ T-cell expansions (78–81). Moreover, TGF-β can negatively regulate the adaptive and innate immune systems by inhibiting important immune cells such as NK cells, effector T cells, and antigen-presenting dendritic cells (82). Studies have also shown that TGF-β strongly decreases key antitumor cytolytic contributors such as NKG2D and perforin/granzyme A and B on γδ T cells while upregulating the inhibitory molecule NKG2A (59, 77, 83). Finally, Beatson et al. observed that cells expanded with TGF-β are cytotoxic against immortalized and non-cancerous cells, posing a possible risk of autoimmunity and toxicity (59). Thus, culturing γδ T cells with TGF-β can possibly enhance γδ T-cell trafficking, but additional studies are needed to understand the possible limitations of this manufacturing strategy.

One of our concerns was whether our SFM-expanded cells retain any migratory properties. To determine whether γδ T-cell trafficking *in vivo* can be manipulated by inducing chemokine expression and taking advantage of corresponding receptors expressed on γδ T cells, we demonstrated that γδ T cells express high *CCR2* mRNA and CCR2 protein. MSCs cultured with IFNγ have increased secretion of the chemokines CCL2 and CCL7, compared to secretion from non-primed MSCs. CCL2 and CCL7 are two chemokine ligands for the CCR2 chemokine receptor (64, 65). Therefore, we sought to examine whether CCR2^+^ γδ T cells can migrate to CCL2-secreting cells *in vivo*. Using intraosseous injections and a human neuroblastoma *in vivo* model, which we previously established (41, 46), it was shown that γMSCs significantly recruit γδ T cells compared to MSCs or a placebo, illustrating that migration of *ex vivo*, SFM-expanded cells can, indeed, be manipulated based on their chemokine receptor profile.

Though a limitation of this study and other murine studies employing human γδ T cells is the lack of long-term *in vivo* persistence, this can be countered using strategies of multiple cell infusions, which have already been clinically tested and well-tolerated (84, 85). However, if the cells do not migrate to the malignant site, increasing the number of cells per dose or the frequency of dosing will not be useful. Therefore, understanding the mechanisms of migration and basic properties of these cells remains a critical aspect of study and is needed, as these cells are being employed clinically in numerous cancer settings. We and others have consistently i) demonstrated the effectiveness of γδ T cells against various types of cancer, ii) illustrated how well these cells expand *ex vivo*, iii) determined their chemokine receptor expression can be manipulated, and iv) showed that the cells retain high cytotoxic potentials independent of HLA class II presentation. Still, we have a fundamental lack of understanding of human γδ T-cell migration *in vivo*, particularly in standard animal models of cancer. We identified important features of γδ T-cell migration *in vivo*, which we think can be useful for future studies. Based on these findings, it is expected that other chemokine relationships of importance can be leveraged to direct γδ T-cell migration *in vivo*, providing additional therapeutic targets, including malignant and non-malignant diseases.

## Data availability statement

The datasets presented in this study can be found in online repositories. The names of the repository/repositories and accession number(s) can be found below: NCBI Sequence Read Archive (SRA) [BioProject ID PRJNA1014486].

## Ethics statement

The studies involving humans were approved by The Emory University Institutional Review Board (IRB). The studies were conducted in accordance with the local legislation and institutional requirements. The participants provided their written informed consent to participate in this study. The animal study was approved by Emory University Institutional Animal Care and Use Committee. The study was conducted in accordance with the local legislation and institutional requirements.

## Author contributions

KP: Conceptualization, Formal analysis, Investigation, Software, Validation, Visualization, Writing – original draft, Writing – review & editing. GB: Conceptualization, Investigation, Validation, Visualization, Writing – review & editing. RB: Conceptualization, Formal analysis, Investigation, Validation, Visualization, Writing – review & editing. AB: Conceptualization, Investigation, Validation, Visualization, Writing – review & editing. AS: Conceptualization, Investigation, Software, Validation, Writing – review & editing. EF: Writing – review & editing, Data curation, Formal analysis. KK: Writing – review & editing, Data curation, Formal analysis. BP: Resources, Writing – review & editing, Conceptualization, Investigation, Validation. EH: Funding acquisition, Project administration, Resources, Supervision, Writing – review & editing. CD: Funding acquisition, Project administration, Resources, Supervision, Writing – review & editing. HTS: Conceptualization, Funding acquisition, Project administration, Resources, Supervision, Visualization, Writing – review & editing.

## Supplementary material

Supplementary Figure 1 γδ T cell phenotype remains unchanged from peripheral blood to bone marrow. Mice were conditioned with 6Gy radiation, 24 hours later injected with γδ T cells, and phenotype markers of live γδ T cells were assessed by flow cytometry. Representative tSNE plots from one of the three biological replicates show protein distribution in each cell population.

Supplementary Figure 2 Human γδ T cells do not migrate to mouse bone marrow despite the presence of human hematopoietic-derived cell lines. NSG mice were inoculated via tail vein with 5e6 CMK-luciferase cells (n = 3) or 2e6 Nalm6-luciferase cells (n = 3). In addition to these mice, appropriate control mice were added: no leukemia or γδ T cell control (n = 1) and γδ T cell only (n = 2). **(A)** After 15 days, mice were surveyed via bioluminescent imaging to track cancer engraftment in the bone marrow. Sixteen days post-leukemia inoculation, 1e6 γδ T cells were systemically infused via retro-orbital injection. Twenty-four hours after, **(B)** bone marrow and **(C)** spleen were harvested and flow cytometry was performed to confirm presence of cancer and percentage of γδ T cells. All statistical combinations were not significant via Student’s t test (p> 0.05 = ns). ns, not significant.

Supplementary Figure 3 Busulfan does not cause γδ T cells to enter the bone marrow. Mice were conditioned with 25mg/kg busulfan and 24 hours later were administered with 10e6 CellTrace CFSE-stained blood from BALB/cJ mice; **(A)** blood and **(B)** bone marrow were harvested and assessed for percentage of CFSE-tagged cells (gated on CFSE^+^ TER119^+^ cells) via flow cytometry, n = 4 mice per condition. Statistics analyzed by Student’s t test, and the sample mean is denoted with a black line.

Supplementary Figure 4 γδ T cells do not express CXCR4 despite differences in expansion methods. **(A)** Histogram of four different CXCR4 flow cytometry antibody clones tested with serum-free-expanded γδ T cells; the Nalm6 cell line was used as a positive control. Peripheral blood mononuclear cell samples from four individual healthy donors were selected for γδ T cells expanded in serum- or serum-free media. The following parameters were analyzed via flow cytometry: **(B)** percentage natural killer cells (CD3^-^ CD56^+^ cells), **(C)** percentage CD16^+^ γδ T cells (gated on CD3^+^ γδTCR^+^), **(D)** percentage CD56^+^ γδ T cells (gated on CD3^+^ γδTCR^+^).

Supplementary Figure 5 γδ T cells express CCR2, and γMSCs secrete CCL2. **(A)** RNA-seq performed on two biological replicates of γδ T cells isolated from two different PBMC donors; genes were ranked, log2(normalized counts) were calculated, and *CCR2* highlighted in red. **(B)** Graph of ELISA of CCL2 secretion by MSCs or γMSCs every 24 hours for 72 hours, n = 3 biological replicates, and each biological replicate is an average of two technical replicates. Statistics analyzed by one-way ANOVA with *post hoc* (p< 0.05 = *, p< 0.01 = **, p< 0.001 = ***). The sample mean is denoted with a black line. **(C)** Graph of transwell migration assay of γδ T cell migration to γMSCs treated with CCL2 antibody, n = 3 biological replicates, and each biological replicate is an average of two technical replicates. Statistics analyzed by one-way ANOVA with *post hoc* (p<0.05 = *); the sample mean is denoted with a black line.
