# Supplementary material for: Directing the migration of serum-free, ex vivo-expanded Vγ9Vδ2 T cells
Source: Front Immunol. 2024 Feb 29;15:1331322. doi: 10.3389/fimmu.2024.1331322 (PMC10937339; doi:10.3389/fimmu.2024.1331322)

Supplementary Figure 1: γδ T cell phenotype remains unchanged from peripheral blood to bone marrow.

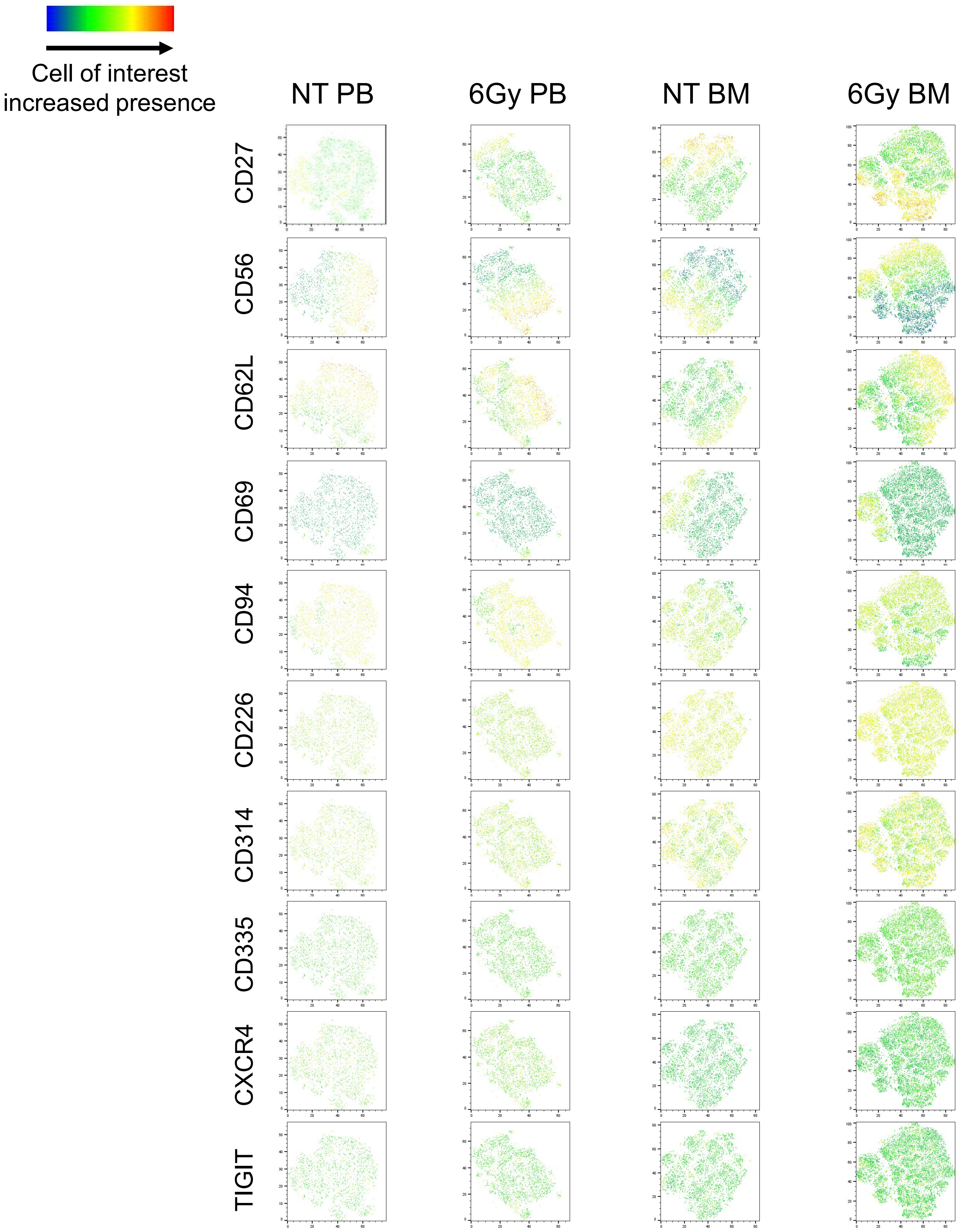

Supplementary Figure 2: Human γδ T cells do not migrate to mouse bone marrow despite the presence of human hematopoietic-derived cell lines.

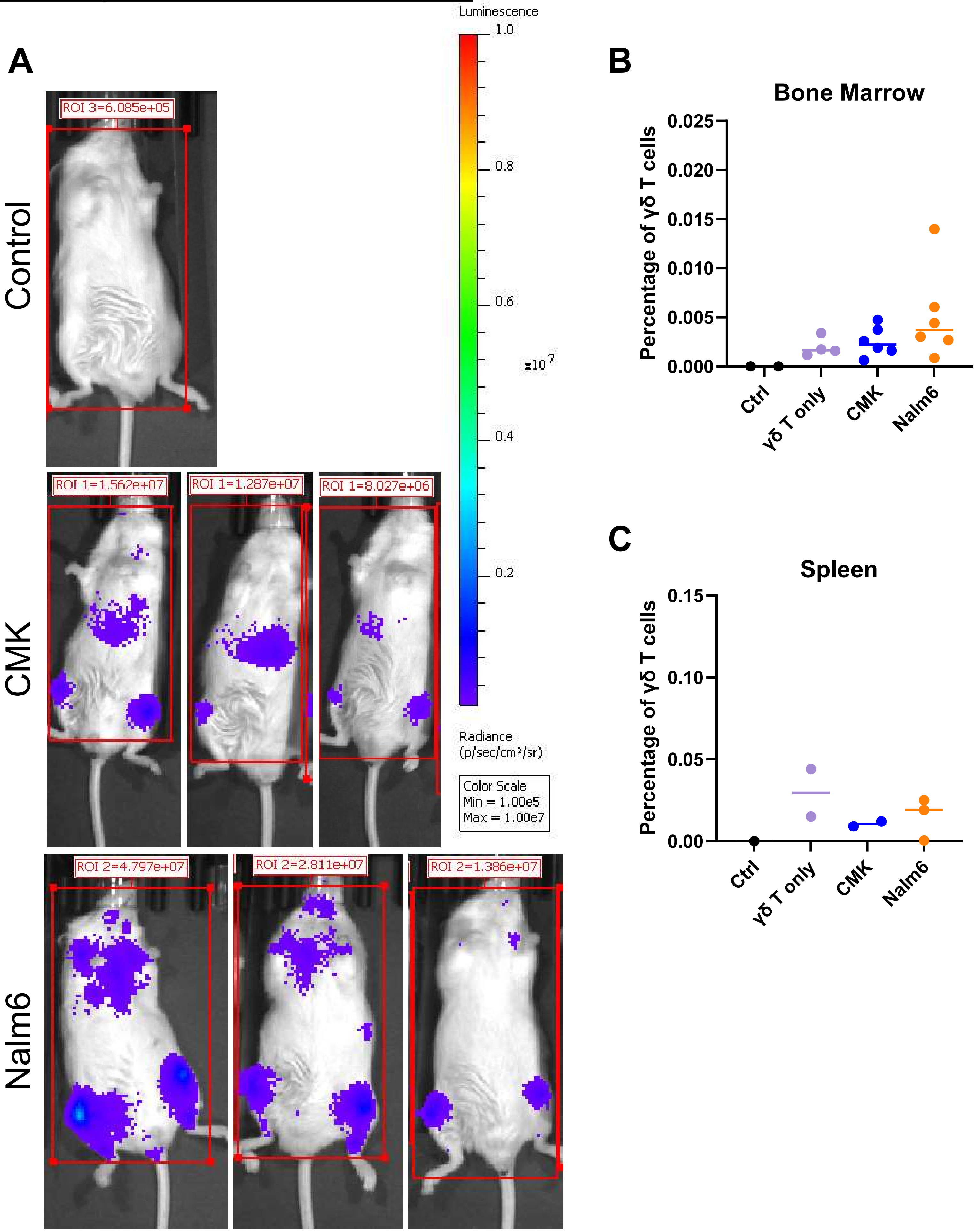

Supplementary Figure 3: Busulfan does not allow γδ T cells to migrate into the bone marrow.

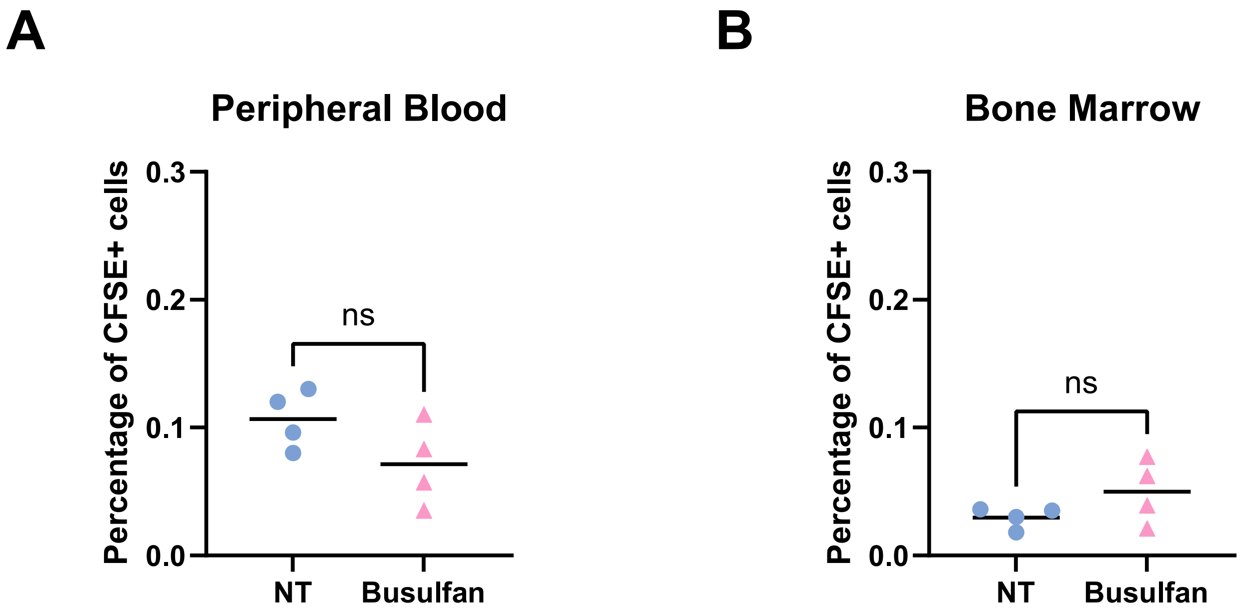

Supplementary Figure 4: γδ T cells do not express CXCR4 despite differences in expansion methods.

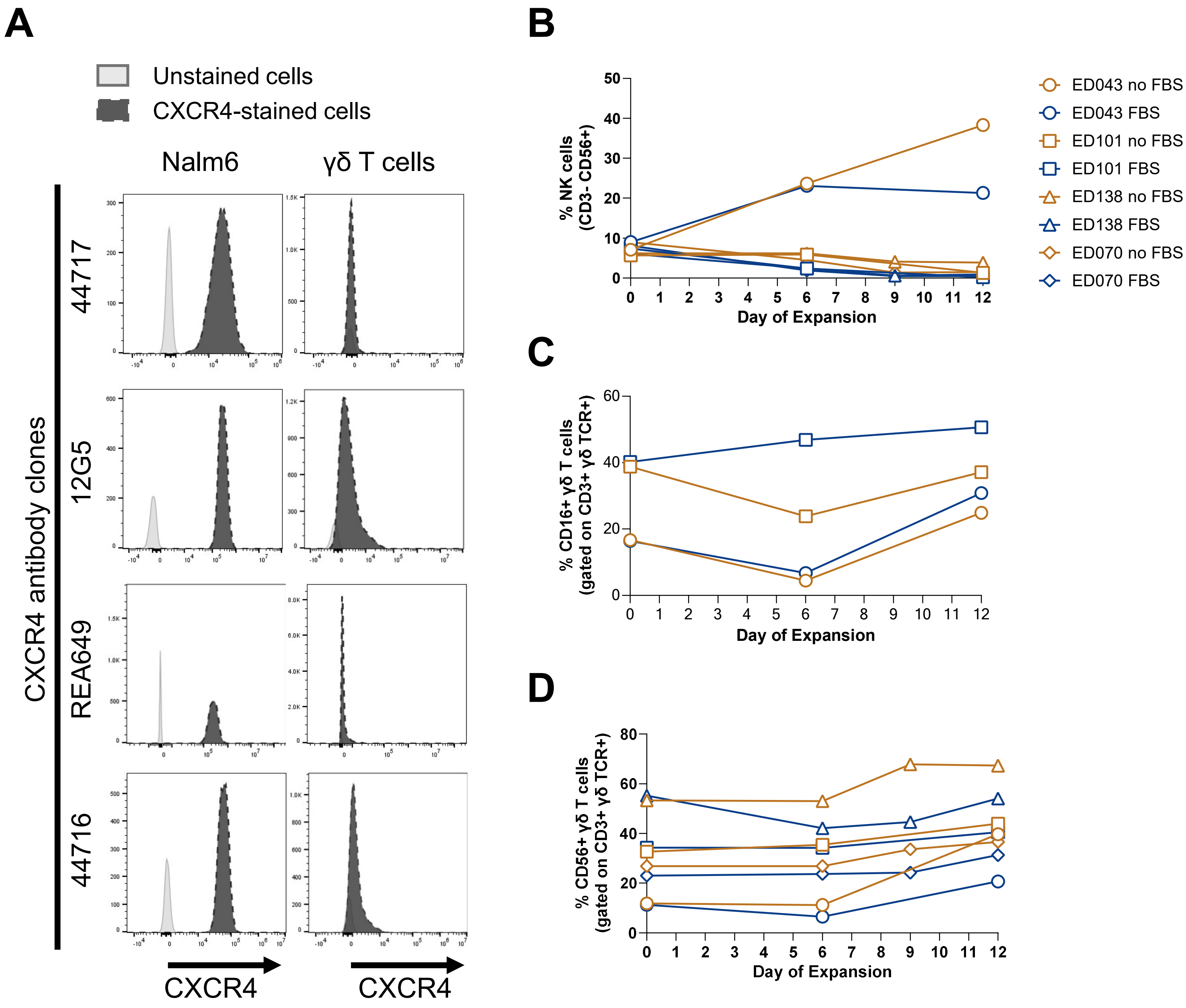

Supplementary Figure 5: γδ T cells express CCR2, and γMSCs secrete CCL2.

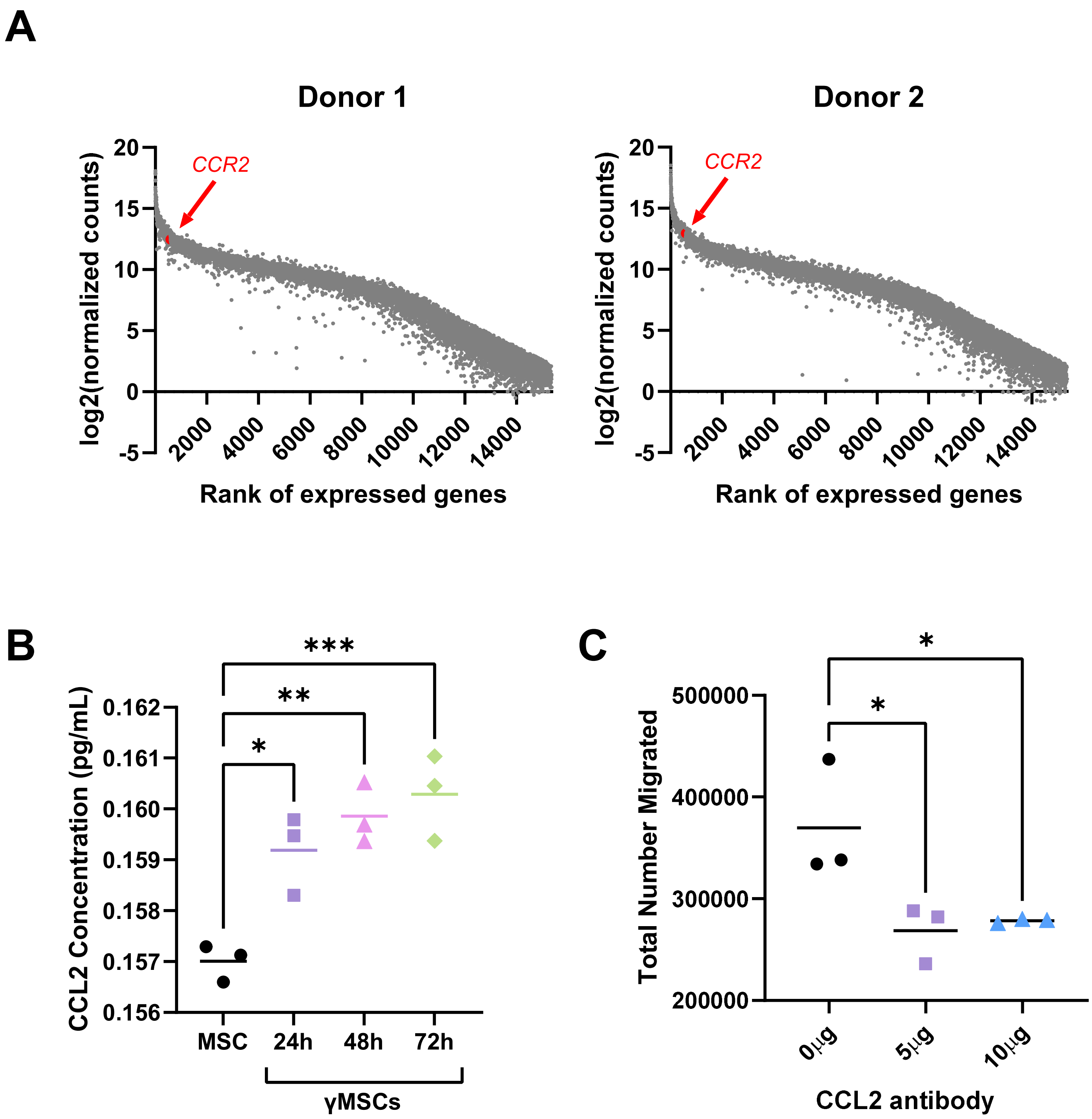

Supplement: Supplementary Figure 1 — γδ T cell phenotype remains unchanged from peripheral blood to bone marrow. Mice were conditioned with 6Gy radiation, 24 hours later injected with γδ T cells, and phenotype markers of live γδ T cells were assessed by flow cytometry. Representative tSNE plots from one of the three biological replicates show protein distribution in each cell population. [file Image_1.pdf]
